# Modulatory effect of olanzapine on SMIM20/phoenixin, NPQ/spexin and NUCB2/nesfatin-1 gene expressions in the rat brainstem

**DOI:** 10.1007/s43440-021-00267-7

**Published:** 2021-04-29

**Authors:** Artur Pałasz, Piotr Żarczyński, Katarzyna Bogus, Kinga Mordecka-Chamera, Alessandra Della Vecchia, Jakub Skałbania, John J. Worthington, Marek Krzystanek, Małgorzata Żarczyńska

**Affiliations:** 1 Department of Histology, Faculty of Medical Sciences in Katowice, Medical University of Silesia, ul. Medyków Street 18, 40-752 Katowice, Poland; 2 Department of Clinical and Experimental Medicine, Section of Psychiatry, University of Pisa, 67, Via Roma, 56100 Pisa, Italy; 3 Division of Biomedical and Life Sciences, Faculty of Health and Medicine, Lancaster University, Lancaster, LA1 4YQ UK; 4 Clinic of Psychiatric Rehabilitation, Department of Psychiatry and Psychotherapy, Faculty of Medical Sciences in Katowice, Medical University of Silesia, ul. Ziolowa 45/47, 40-635 Katowice, Poland

**Keywords:** Olanzapine, Phoenixin, Spexin, Nesfatin-1, Brainstem

## Abstract

**Background:**

Phoenixin, spexin and nesfatin-1 belong to a family of newly discovered multifunctional neuropeptides that play regulatory roles in several brain structures and modulate the activity of important neural networks. However, little is known about their expression and action at the level of brainstem. The present work was, therefore, focused on gene expression of the aforementioned peptides in the brainstem of rats chronically treated with olanzapine, a second generation antipsychotic drug.

**Methods:**

Studies were carried out on adult, male Sprague–Dawley rats that were divided into 2 groups: control and experimental animals treated with olanzapine (28-day-long intraperitoneal injection, at dose 5 mg/kg daily). All individuals were killed under anesthesia and the brainstem excised. Total mRNA was isolated from homogenized samples of both structures and the RT-PCR method was used for estimation of related SMIM20/phoenixin, NPQ/spexin and NUCB2/nesfatin-1 gene expression.

**Results:**

Long-term treatment with olanzapine is reflected in qualitatively different changes in expression of examined neuropeptides mRNA in the rat brainstem. Olanzapine significantly decreased NPQ/spexin mRNA expression, but increased SMIM20/phoenixin mRNA level in the rat brainstem; while NUCB2/nesfatin-1 mRNA expression remained unchanged.

**Conclusions:**

Olanzapine can affect novel peptidergic signaling in the rat brainstem. This may cautiously suggest the presence of an alternative mode of its action.

## Introduction

Neuropeptides play an important role in numerous brainstem functions. So far, the best characterized is neuropeptide Y (NPY), consisting of 36 amino acids [1] with homology to the pancreatic polypeptide (PP) and the YY peptide [2]. The important influence of NPY on many physiological functions, such as food intake, blood pressure, sexual activity and circadian rhythm has previously been established [3]. In the brainstem, there is also a distinct expression of neuropeptide S (NPS), a 20-amino acid peptide which selectively activates the G protein coupled NPSR receptors [4]. NPS activity is manifested by reducing the level of anxiety and appetite, while on the other hand, it increases mobility, fatigue and hypersexual changes [5]. NPS has also been shown to contribute to addiction development and circadian rhythm control [6, 7]. In the case of NPS, the stimulatory effect of antipsychotic drugs on expression level of this neuropeptide has also been demonstrated in the rat hypothalamus [8].

The newly discovered but still understudied multifunctional neuropeptides, phoenixin (PNX), nesfatin-1 and spexin (SPX) as the potential modulators of several brainstem functions seem to be especially worth mentioning. PNX is a regulatory neuropeptide identified seven years ago by Gina Yosten and colleagues [9], functioning in two molecular forms as PNX-14 and PNX-20, resulting from post-translational processing of the SMIM20 prohormone. Immunohistochemical studies of rodent brain revealed PNX expression in spatially restricted to populations of hypothalamic and brainstem neurons [10]. A significant population of kisspeptin neurons of the arcuate nucleus are characterized by PNX expression [11] and these cells are the source of axons reaching GnRH neurons in the preoptic hypothalamus [12]. Recent studies have shown that PNX binds to the metabotropic GPR173 receptor present in numerous hypothalamic neurons [13]. GPR173, also known as SREB3, represents a unique super-conservative group of regulatory molecules (SREBs), present both in the CNS and in peripheral organs [14]. Stimulation of effector neurons by PNX binding to the GPR173 receptor triggers the cAMP/protein A kinase pathway through CREB and possibly C/EBP-β and/or Oct-1 [13]. Signaling allows PNX to regulate pituitary gonadotropin secretion by modulating expression of the gonadoliberin receptor (GnRH-R). It has been suggested that PNX sensitizes pituitary cells to other releasing factors without directly stimulating their exocytosis. PNX may, therefore, be a novel hypothalamic regulatory factor that stimulates the action of gonadotropic cells in the pituitary gland. Hypothetically, PNX may also activate kisspeptin neurons in an autocrine manner and/or through association with other PNX-expressing cells [13]. It was also observed that externally administered PNX may preferentially inhibit visceral pain as compared to thermal pain, potentially through other signaling pathways such as the recently reported signal transduction mechanism utilizing the MAPK/ERK pathway. Moreover, the majority of PNX neurons in the rat hypothalamus coexpress nesfatin-1 [15], suggesting further potential interactions with other novel neuropeptides.

It has been reported that immobilization stress significantly increased the expression of PNX in the hypothalamic neurons and the rat brainstem due to the stimulation of their activity (via increased expression of c-Fos), suggesting that the PNX neuropeptide is involved in the response to external stress factors [16]. PNX also influences the excitability of solitary nucleus neurons, and its action depends on environmental stress factors and glucocorticoids [17]. PNX stimulates the secretion of vasopressin and is an element of neuronal loops that regulate the endocrine and behavioral mechanisms of electrolyte homeostasis in the body [18]. Recently, studies have also suggested a neuroprotective effect of PNX-20 dependent on SIRT1 and the participation of this neuropeptide in the modulation of inflammatory reactions in the CNS [19].

The neuropeptide nesfatin-1 molecule consists of 82 amino acid residues and is formed by post-translational hydrolysis of nucleobindin-2 (NUCB2). The amino acid sequence of nesfatin-1 is highly conserved among vertebrates [20], with the expression of the nucleobindin-2 gene confirmed in the brain and adipose tissue. In the brain, expression occurs mainly in the nuclei of the hypothalamus and the brainstem [21]. The main physiological effect of nesfatin-1 is inhibition of food intake. Direct delivery to the rat brain causes an anorexigenic effect for about 6 h, and administration of nesfatin-1 neutralizing antibodies causes the opposite effect—an increase of appetite, an action independent of leptin. It has been proven that the hydrolysis of NUCB2 occurs physiologically in the brain, since the presence of nesfatin-1 and the N-terminal fragment of nucleobindin-2 has been confirmed in cerebral fluid [20]. It has also been suggested that, in addition to its food-inhibiting effect, nesfatin-1 may be involved in anxiety reactions [22]. Its intraventricular administration causes stress symptoms in rats and produces an increase in the levels of adrenocorticotropic hormone (ACTH) and corticosterone in the blood plasma [23]. The penetration of nesfatin-1 across the blood–brain barrier has been confirmed [24] and the expression of NUCB2 gene in the periventricular and supraoptic nucleus can be modulated by fasting-refeeding [25]. Considering the influence of antipsychotic drugs on the expression of the NUCB2/nesfatin-1 gene, it should be mentioned there has been confirmed decreases in its expression after long-term use of the classic drug haloperidol [26].

Spexin (SPX), encoded by the *C12ORF39* gene, is a highly conserved 14 amino acid regulatory neuropeptide and was discovered in 2007, thanks to bioinformatics techniques [27]. SPX is a natural ligand for GalR2/3 galanin receptors [28] and research has confirmed that NPQ/SPX gene expression in the brain and peripheral tissues in both humans, mice, fish and rats. Expression in the central nervous system concerns such regions as the cerebral cortex, the hippocampus and the brainstem [29]. The multiple sites of secretion suggest that SPX has multiple physiological functions, e.g., its effect on reproduction and appetite in fish has been confirmed; SPX causes a decrease in luteinizing hormone secretion [30] and suppresses the appetite [31]. It has also been noticed that an increase in insulin levels, considered to be a determinant to satiety, increases the expression of the NPQ/spexin gene in the brain and liver [32]. The disadvantage of these reports, which may limit their reference to human physiology, is the fact that the research was carried out mainly on fish. In mammals, the confirmed functions of SPX are inhibition of the proliferation of adrenal cortex cells [33], regulation of nociceptive responses, modulation of cardiovascular reactions [34], regulation of gastric contraction induction and, importantly, an effect on weight loss [35].

Olanzapine, representing the group of thienobenzodiazepine derivatives, is an atypical antipsychotic drug with proven efficacy in the treatment of schizophrenia and related psychoses. Its structural and pharmacological properties are in many ways similar to another clinically important drug from this group—clozapine. Compared to classic antipsychotic drugs, which are represented by haloperidol or chlorpromazine, olanzapine has a much more favorable tolerance profile [36]. Olanzapine has high affinity for serotonergic 5-HT_2A_ receptors and moderate affinity for dopaminergic D_1_, D_2_, D_4_, serotonergic 5-HT_2C_, 5HT_6_, 5HT_7_, histamine, α1-adrenergic and muscarinic receptors [37]. Studies have shown that olanzapine is as effective as haloperidol in combating positive symptoms of schizophrenia, but more effective in reducing negative symptoms [38].

So far, numerous studies have been conducted on the influence of olanzapine on the level of gene expression of many neuropeptides, while some of them may significantly contribute to the side effects of treatment with this antipsychotic drug. The aim of the study was to investigate the effect of chronic administration of olanzapine on the expression of SMIM20/phoenixin, NPQ/spexin and NUCB2/nesfatin-1 genes in the rat brainstem.

## Materials and methods

### Animals

The studies were carried out on adult (5 months old, 190–210 g) male Sprague–Dawley rats from Medical University of Silesia Experimental Centre housed at 22 °C with a regular 12/12 light–darkness cycle with access to standard Murigran chow and water ad libitum. In addition, the animal body weight was measured weekly without an estimation of food intake. All experimental procedures were approved by Local Bioethical Committee at the Medical University of Silesia (agreement no. 36/2012) and were conducted in a manner consistent with NIH Guidelines for Care and Use of Laboratory Animals.

### Drug administration and mRNA collection

**Fig. 1:**
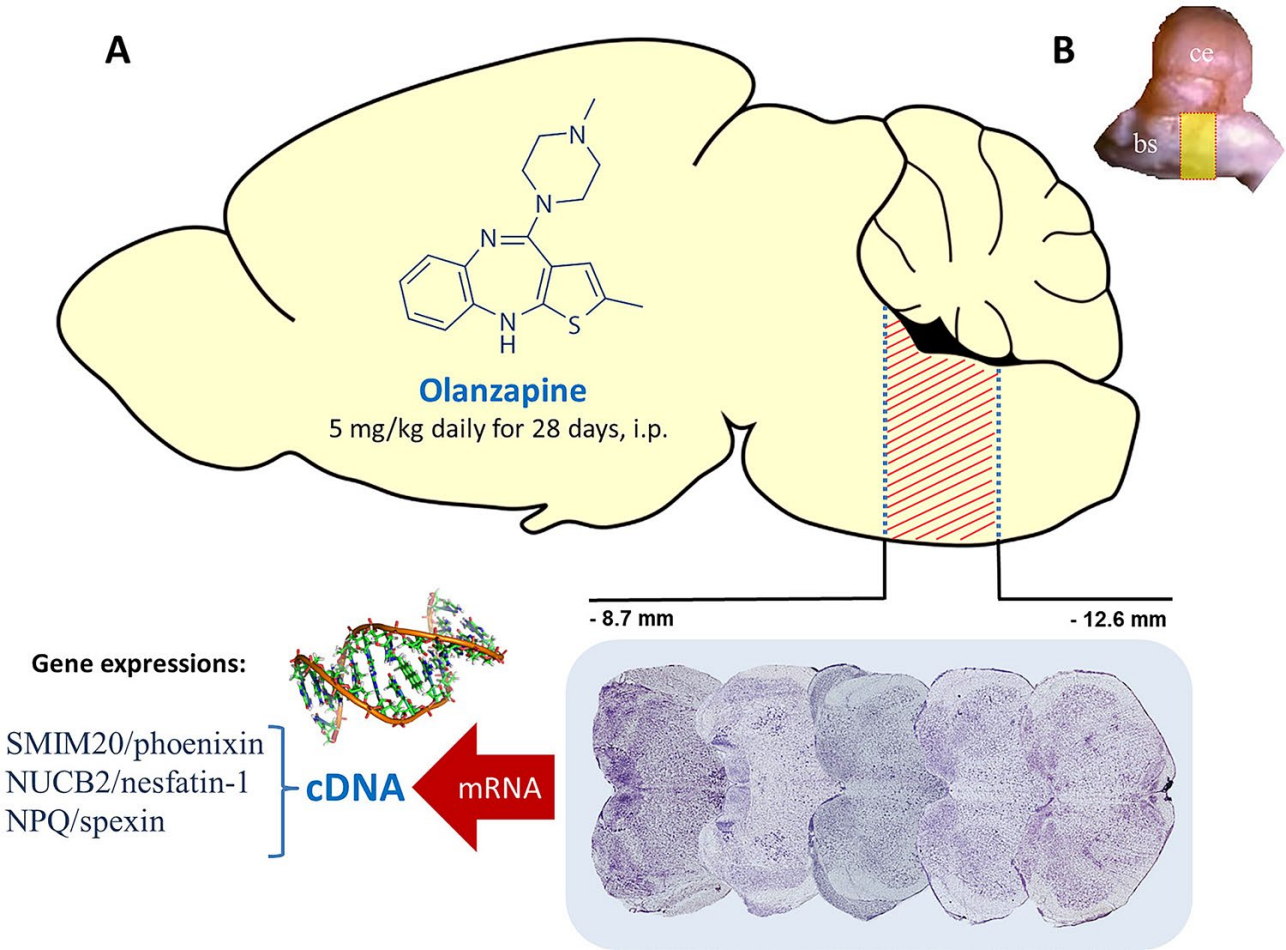
Schematic representation of the experimental method (**a**). The brainstem segments (− 8.7 to − 12.6 mm from bregma, **a**, **b**) were excised, total mRNA was isolated and the Real-Time PCR method was used for estimation of related SMIM20/phoenixin, NPQ/spexin and NUCB2/nesfatin-1 gene expressions. Tissue samples contained the main brainstem nuclei including aminergic and peptidergic perikarya. *bs* brainstem, *ce* cerebellum. Structural figures based on modified brain sections taken from the standard Paxinos and Watson The Rat Brain Atlas [47]

Two groups of animals received olanzapine (5 mg/kg/day) or control saline vehicle by intraperitoneal injection for 4 weeks. 24 h after the last drug administration, rats were quickly anaesthetized with isoflurane and killed. Total mRNA was extracted from excised brainstem sections (each sample was taken from one individual, segments from − 8.7 to − 12.6 mm from bregma, Fig. 1.) via the phenol–chloroform method using Trizol™.

### RT-PCR

Collected mRNA samples were transcribed into cDNA during incubation in buffered solution of reverse transcriptase MMLV-RT with RNAsine, oligo-dT and mix of nucleotides at 42 °C for 60 min using Veriti 96 Well DNA Thermal Cycler (Applied Biosystems, Foster City, CA, USA). After that, Quantitative Real-Time PCR reaction (qPCR) was performed by FastStart SYBR Green Master in a Light Cycler 1 96 (Roche, Switzerland). Glyceraldehyde-3-phosphate dehydrogenase (GAPDH) was chosen as a standard internal reference gene. Primer sequences; GAPDH: Forward: 5′-GTGAACGGATTTGGCCGTATCG-3′, Reverse: 5′-ATCACGCCACAGCTTTCCAGAGG-3′, NUCB2/nesfatin-1 (NM_021663.2): Forward: 5′ TTTGAACACCTGAACCACCA 3′, Reverse: 5′ TGCAAACTTGGCTTCTTCCT-3′; SMIM20/phoenixin (NM_001134639.2): Forward: 5′-GTGGTCTGATCCGTTTGGCA-3′, Reverse: 5′-CTATCACAAGGGCTCTGGCT-3′. For NPQ/spexin (NM_001083933.3) analysis, cDNA was amplified using the TaqMan Gene Expression Assay Spexin (Rn01749065_m1, Applied Biosystems) and TaqMan Gene Expression Master mix (4369016, Applied Biosystems). Optimal hybridization temperature was established according to a gradient PCR and was 50 and 59 °C.

### Statistics

Statistical analyses were performed using Statistica (Systat Software, San Jose, CA, USA). Data are presented as mean ± SEM. Mean differences between experimental groups were analyzed using one-way ANOVA followed by Tukey’s post hoc test. Differences were considered statistically significant at *p* ≤ 0.01.

## Results and discussion

PNX, SPX and nesfatin-1 have diverse and multidirectional physiological actions in the brain that result in hormonal, biochemical and even behavioral changes. The presented initial study investigated the effect of chronic administration of olanzapine, an atypical antipsychotic drug, on the expression level of the genes of the neuropeptides (spexin) or their precursors (NUCB2/nesfatin, SMIM20/phoenixin). Noteworthy, some of the side effects that occur in people taking olanzapine correspond to some aspects of the physiological activity of the regulatory peptides examined. To determine whether olanzapine affects the expression level of aforementioned neuropeptides in the rat brainstem, the level of transcript mRNA was determined after 28 days of administration of the neuroleptic (chronic pharmacological model). Significant changes in the expression of their genes (or the genes of their precursor proteins) were shown in the case of two of the three examined neuropeptides—SMIM20/phoenixin and spexin. The expression level of the NUCB2/nesfatin-1 gene was not changed.

**Fig. 2:**
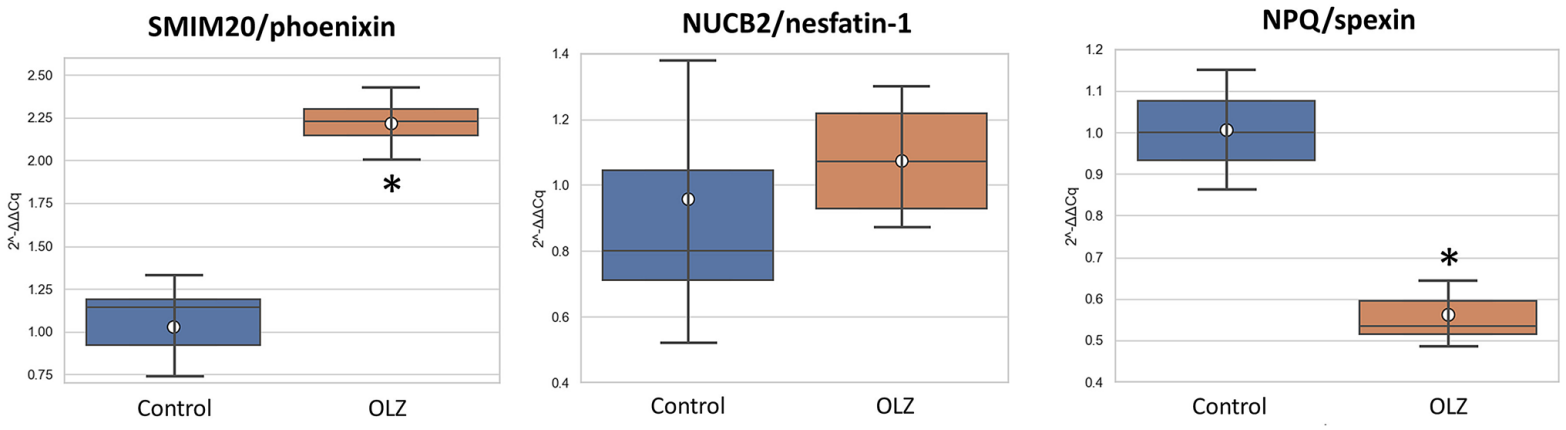
Relative mRNA expression of SMIM20/phoenixin, NUCB2/nesfatin-1 and NPQ/spexin in the rat brainstem after long-term olanzapine administration. Number of animals per group (*n* = 5). Glyceraldehyde-3-phosphate dehydrogenase (GAPDH) was used as a reference gene. Values are expressed as means ± SEM. Differences between experimental groups were analyzed using one-way ANOVA followed by Tukey’s post hoc test and they were considered significant at *p* ≤ 0.01 (asterisks)

Statistical analysis revealed that treatment with olanzapine significantly increased the level of the SMIM20/phoenixin gene expression (Fig. 2; *F* = 72.350; *p* = 0.000) and the difference was 201% of control. Despite the immunohistochemical confirmation of co-expression of PNX and nesfatin-1 in the hypothalamic neurons [15], the expression level of the NUCB2/nesfatin-1 gene did not change significantly following treatment (Fig. 2; *F* = 0.219; *p* = 0.997). Perhaps, therefore, the nesfatin-1 neurons with PNX co-expression are characteristic of the hypothalamus and are not present in the brainstem nuclei. It may also indicate that nesfatin-1 is not involved in the molecular mechanism of olanzapine action at the brainstem level. Probably the NUCB 2/nesfatin-1 gene is characterized by a high degree of stability in the cells of the brainstem. On the other hand, it is worth emphasizing that chronically administered haloperidol caused a dramatic increase in the expression of neuropeptide S (NPS) mRNA in the brainstem under these conditions [39]. NPS is an endogenous anxiolytic factor, which suggests the effect of haloperidol on anxiety and stress responses in an animal model, which, must be confirmed by further molecular and behavioral studies. Studies by Bloem et al. [40] determined the expression of NUCB2/nesfatin-1 mRNA in the Edinger–Westphal (EW) nucleus of males who died as a result of suicide, providing extremely intriguing data. NUCB2 mRNA was significantly elevated as compared to the control group, while in female cases it never reached the control level seen in humans [40]. This original and intriguing study suggests the involvement of nesfatin-1 in the brainstem in processes related to the pathogenesis of autoimmune and depressive behaviors.

The effect of PNX on stress responses in rodents has been recently observed [41], with targeted infusion of PNX-14 into the hypothalamus, but not into the amygdala, reducing the anxiety behavior of adult mice in the open field and elevated plus maze tests. This effect was counteracted by the administration of a GnRH receptor antagonist (cetrorelix), while the blockade of oxytocin receptors by atosiban did not affect the described behavioral effects. Thus, the anxiolytic effect of PNX-14 is based on the GnRH signaling pathway and is independent of oxytocin [41]. Clinical trials have also shown that the concentration of PNX in plasma was negatively correlated with anxiety (assessed via neuropsychiatric methods) in obese men [42]. Injection of PNX-14 into the lateral ventricles and the hippocampus facilitates memory consolidation in rats. This neuropeptide may minimize the memory deficits induced by the amyloid-β1-42 peptide [43]. In geriatric patients with mild cognitive impairment (MCI) but not Alzheimer’s disease, mean serum PNX levels were negatively correlated with logical memory. Thus, fluctuations in PNX levels may be a potential prognostic marker of the initial phase of MCI [44]. The aforementioned studies may confirm the potential cognitive effects of the influence of olanzapine on the levels of PNX mRNA level in the CNS.

Our study also reports that olanzapine administration decreased the NPQ/spexin gene expression (Fig. 2; *F* = 59.377; *p* = 0.000) and the difference was 55% of control. Several reports show that SPX inhibits food intake [31], furthermore, in the state of satiety, high levels of NPQ may be the result of increased insulin production [32]. Thus, if chronic administration of olanzapine causes a decrease in the expression level of NPQ/spexin mRNA and potentially of the neuropeptide itself, it could theoretically lead to an increase in appetite. It cannot be ruled out that the decrease in the level of SPX in the brainstem belongs to the multi-element set of neurochemical causes of one of the major side effects of neuroleptics, which is weight gain. Moreover, the role of brainstem neurons in the regulation of energy homeostasis is not entirely clear, especially since we do not know which populations were a potential target of olanzapine. The answer to this question may be provided by immunohistochemical studies of the nuclei of the brainstem in animals treated with neuroleptics. These determinations should be performed analogously to previous studies on the effect of escitalopram on the level of SPX expression [45], which showed a significant decrease in the neuropeptide expression in the rat hypothalamus. It is worth noting here that the physiological effect of olanzapine observed in the brainstem turned out to be identical to that of escitalopram (an antidepressant) in the hypothalamus. Our results, however, do not correspond with increased NPQ/spexin mRNA expression in the amygdala of rats exposed to the classic antipsychotic drug chlorpromazine [46].

## Conclusions

The SPX and PNX signaling pathways of rat brainstem neurons may be sensitive to the chronic effects of olanzapine. Olanzapine is a factor that promotes the expression of the SMIM20/phoenixin gene in rat brainstem cells with features of an inhibitor of spexin expression. The expression of the NUCB2/nesfatin-1 gene in rat brainstem neurons is relatively stable under the operating conditions of this neuroleptic. One possible alternative mechanism of the pharmacological activity of olanzapine in the animal model is its effect on the expression level of the NPQ/spexin and SMIM20/phoenixin genes in the brainstem. However, this conclusion should be treated with great caution, because the expression level of the peptide itself was not determined and the size of the research group was limited. The work touched upon neurochemical issues that are important from the neuropharmacological point of view, and so far have not been investigated. It is undoubtedly a starting point to a broader examination at novel mechanisms of side effects of antipsychotic drugs.
